# Twelve tips for personal advisers to undergraduate medical students

**DOI:** 10.15694/mep.2018.0000198.1

**Published:** 2018-09-05

**Authors:** Margaret Bunting

**Affiliations:** 1 University of East Anglia

**Keywords:** adviser, advisee, undergraduate, student, medical education, support, mental wellbeing, Personal Adviser, academic support, professionalism development, mentoring, career advice, elective abroad, resilience

## Abstract

This article was migrated. The article was marked as recommended.

Most Higher Educational institutes have a system whereby a member of staff is identified as the student’s personal adviser. The adviser’s role is to offer advice and guidance, commonly for the full duration of the student’s program. This is an important responsibility as it can involve a complex mixture of advice relating to academic support, pastoral concerns and health issues, as well as professionalism development. Despite the adviser being a key person in delivering the institution’s duty of care, there is very little training and guidance for the teaching staff themselves when undertaking this role. Different Universities will use different titles for such a role and when using the term ‘personal adviser’ within this paper, the content can be transferable to any tutor whose role is to advise on a one to one basis. The aim of this paper is to offer suggestions and strategies for tutors to consider in order to support individual students in a holistic manner, and generate a discussion on the challenges to achieving effective individual support in a demanding higher education environment.

## Introduction

Academic advising offers a unique teaching opportunity for ongoing, one-to-one interaction with an academic representative within a higher educational institution. As a result of this opportunity, academic advising has the power to be at the very core of successful institutional efforts to educate ( Tinto, 1999). Ultimately, academic advising can assist the University with student retention, high academic achievement as well as high student satisfaction rating. In order to gain meaning from an adviser/advisee meeting, however, there needs to be an element whereby the students learn to be advised, and the adviser learns to develop the skills of advising.

## Development of my recommendations

My aim is to give tips to support academics in the development of their role as an adviser and enhance the student’s experience at university along with reaching their full potential. These tips are synthesised from my ten-years experience as a personal adviser and senior adviser within a UK medical school, along with my doctoral research in student perceptions, and discussions amongst respected colleagues within my university, with other medical schools, and at global academic advising conferences.

## Tip 1 - Prepare for each advising session

Each session should be planned in terms of its timing, the agenda for the meeting, and in being prepared for any likely issues that the student may have. Planning should initially ensure you have allowed adequate and uninterrupted time with the student, and take into account time to make notes following the session.

Before the agreed session, take time to get up to speed with your advisee’s academic history, exam grades, the previous meeting discussion topics and any previous goals set. This will help you consider an appropriate agenda for the forthcoming meeting. What issues or concerns do you have that you want to raise with the student? Your advisee’s curriculum should also be taken into account in order to see what is coming up in the near future, and anticipate any issues the student may currently have.

On arrival, students should be able to add to the agenda. What issues or concerns has the student currently got that they would like to discuss? A good session is one in which both parties have a clear understanding of the structure of the session and that it is inclusive.

## Tip 2 - Engage with any early alert system that is in place within your faculty

Early alert systems offer a proactive approach to reaching out to students who are ‘at risk’, be they academic, pastoral, health or professional issues. The senior adviser within your faculty will know of any early alert system, and the personal adviser is best placed to see the student in the first instance. If you know the trigger for any proactive involvement from senior level staff, you can monitor your advisee, and encourage them to see you before this trigger is activated. Your aim is to intervene before small issues become big. For example, attendance concerns can escalate to professionalism concerns, yet there may be a health issue causing the underlying problem. Young adults managing possibly new mental health issues can be slow to recognise the need for professional support, and your guidance here can be valuable in developing appropriate self-management. Schools frequently send out information about forthcoming activities or deadlines before students request or realise they need it. Following up on such announcements, directly with your adviser, can ensure they understand the relevance of receiving such information.

## Tip 3 - Be proactive

Consider developing your own early alert system. Your aim is to intervene at the first indication of difficulties in order to motivate a student to seek help. Students are often reluctant to self-refer. Consider critical times when you should connect with your advisees. For example, the first two weeks of them starting university, the week before major exams, identify any long spells of study where fatigue and/or burnout may be an issue.

In order to reach out to students during these times I suggest you consider a communications calendar. By this I mean determine the times when it would be prudent to reach out to targeted students. In order to do this, it is worth discussing with the senior adviser what the potential stumbling blocks may be in any given year of study. Also, add the dates to reach out to all advisees to cover appropriate issues such as career and elective planning.

## Tip 4 - Build a professional mentoring relationship with your advisee

Students, whilst informed that they have an adviser, often do not know what the role of the adviser actually is. On your first meeting, explain how they can contact you, and be realistic about the time frame they can expect you to return their call. Consider and discuss with your advisee whether you both make use of phone, email, or Skype as well as in-person appointments. Nonetheless, there should be an emphasis on personal individualised contact in order for you to get to know your advisee, and offer an environment for them to disclose any sensitive information they may want to talk through. Developing an adviser/advisee relationship that extends beyond the readily observable, such as their exam data, will allow meetings to become meaningful in terms of supporting a student to reach their full potential. Taking time to build a relationship prepares the ground if your student runs into difficulty. At that point, you need to be able to get to the root of your advisee’s academic issues and concerns in order to disseminate information and offer appropriate advice. Building a safe learning environment is a concept that is well understood, and advising is no exception. Students need to feel secure in discussing academic and personal concerns, and the adviser who expresses an active concern for the welfare of every advisee can provide this safe environment. I suggest you aim to understand more than the advisee’s academic history. For example, enquire and learn about their family history. International students and first generation students are identified as being at higher risk of falling behind others in their cohort. Students may be primary carers to children or elderly relatives and any absences that a student takes should be carefully explored, making no assumptions that they just ‘didn’t feel like it’. Financial issues may require students to take on paid work as well as study, and exploring the consequences of managing these two demands can be useful. They may consider negotiating fewer working hours leading up to exams. There are many non-academic factors that can affect a student’s ability to reach their full academic potential. It can only be beneficial to explore these with someone who can guide them towards identifying these issues before it is too late.

## Tip 5 - Understand the student’s perspective – advising in context

Student learning is the key focus of the adviser/advisee relationship. Academics may base their evaluation of a student’s overall ability on the student’s academic performance at any given time, and be unaware of any social or personal factors which may be affecting it. Some students can compensate for these distractions with strategies based around successful compartmentalisation, but developing healthy boundaries is really only just beginning to develop in these young adults. Assist the student in this development by showing an interest in their lives and circumstances and help advisees to handle problems proactively before reaching crisis level. They may need support to develop an internal locus of control by recognising the relationship between their own actions and academic success.

Listening to the students’ perspective on their lecturers or curriculum is an opportunity for students to debrief, but also to get a different perspective on a learning situation and who better to offer them this than another lecturer? If students who put a strong emphasis on how well a lecture is delivered, for example, can be encouraged to see a learning opportunity irrespective of the delivery, then they may move in a positive direction and understand the importance of turning up to all lectures.

Student’s who do not feel, initially, that they have a cultural fit with the school need to develop success strategies and skills to help them find their place. Taking a holistic perspective rather than just academic, the adviser can encourage the student to stay motivated. The adviser may be able to put their advisee in touch with a more senior student who can offer to be a supportive role model and mentor.

## Tip 6 - Take an active concern over an advisee’s academic preparation

Students can get the most out of university with good efficient study skills and time management strategies, as well as classroom attendance. Discussing ‘how do you go about learning?’ can be powerful as students identify and critique their personal study habits. Assist in identifying resolvable causes for poor academic performance.

Assisting a student to identify their strengths as well as their weaknesses allows them to consider what learning strategies to keep, and how to regulate and monitor their own progress, without focussing only on the negatives. Students who overstate their academic ability and understate their areas of challenges need particular attention. Self-regulation is key to learning and students need to identify accurately where to focus attention. Students can often feel they are ‘slow’ at learning certain topics, and yet have no benchmark as to whether this is an accurate assessment or not. Such uncertainty can affect their motivation and confidence.

Academic management tasks are often new to a school leaver, yet few courses start with study preparation sessions. Ask the students what their short and long-term goals are and, if they don’t have any, help them to see that such goals would be of benefit. Without goals, there cannot be an action plan. Meeting deadlines and regular class attendance need to be seen as part of any academic action plan.

Asking your advice on the day an assignment needs to be handed in isn’t likely to bring out the best in either of you. Be upfront that your role isn’t to pilot run marking of assignments. If you inform them of this in advance, you can maintain a good relationship. However, you may want to offer feedback on their grammar or the ability to critically analyse. In this situation, offer to give detailed feedback on only one or two paragraphs with the goal that they start to identify if there are any patterns to their errors in the rest of their work. In my experience with students who have had a focus on science subjects, going back to basics on sentence structure may be required. There are some excellent books available to students on this but they are more likely to be motivated to improve if you direct them to the aspect/chapter you have identified as being particularly relevant to their area of weakness.

It is the personal adviser who is often the link to support services offered at university level. Having an awareness of the University’s student support services will assist in decisions on referrals. For example, they may offer time management or stress management workshops.

## Tip 7 - Know when to own and when to refer

Finding what is at the heart of a problem or concern is necessary before recommending appropriate interventions or strategies, and before referring a student to appropriate services that will meet their needs. Student characteristics such as self-esteem, motivation, energy levels and personal factors can impact on a student academically.

To maximise the probability of the student’s academic success may require involving the wider services within the university, and possibly beyond, such as health care professionals. The aim is to avoid ending up in an irreversible and negative situation such as an advisee failing to progress into a more senior year. Students have a right to appeal and if there is a suggestion that success could have been more achievable with appropriate support/treatment the student, quite rightly, can be offered another opportunity. However, this isn’t timely identification and the impact on the student’s confidence could then start to affect their success. Therefore, timely referral to services such as dyslexic screening, or taking a formal break in study for health or personal reasons, is more beneficial in the long term.

## Tip 8 - Include long-term goal setting and career advice into your sessions

Students who feel overwhelmed by the curriculum can feel burnt out and unmotivated. Supporting a student to consider their long-term goals can go a long way in keeping them on track. The adviser can support the student to identify dreams/ideas and start to examine requirements that can lead to some initial goal setting and planning in order for the student to have career readiness. Questions you ask can lead to clarity for a student. For example, asking about their natural strengths, and which classes or curriculum activity they particularly enjoy.

Doctors apply for posts relating to their future career within two years of graduating, so students need to consider their options and develop and enhance their CV in preparation for speciality application. Undertaking additional activities such as research, audits, or undergraduate teaching can be suggested.

## Tip 9 - Consider the impact that placements and elective abroad may have on a student

Are there certain clinical placements that could affect your advisee? Students may find certain ‘triggers’ affect their wellbeing, for example a psychiatric placement for students who themselves have a history of mental health, or a placement such as oncology that may trigger distressing memories. Being aware of any school policy which allows students to ask for personal circumstances to be taken into consideration during placement allocation can support you in your role. Another example could be a need for a student to have a local placement in order that they may continue to access ongoing health treatment. Advice regarding the dress code of a given placement could prove useful, and the placement’s ‘raising a concern’ policy. Students should also consider their own safety especially around returning home after a late shift.

When choosing where to go for their elective, the student should consider factors such as the type of cultural experience that would be most valuable to them. What medical experience would they like to gain? What type of health practice do they want to work in? What are their financial constraints? Another consideration can be whether to go alone or with peers. The advantages of going with someone include an opportunity to talk over the experiences of the day, and feeling potentially less isolated and lonely. Disadvantages include the risk of falling out with your peers in times of stress, alternatively, not feeling a need to build further relationships with new colleagues.

## Tip 10 - Consider building the student’s resilience as part of your role

Personal and family concerns can have a significant impact on students’ wellbeing and their ability to manage a demanding course. An adviser is one of the few staff members who can support an individual student to manage family obligations along with the demands of the course. Encouraging timely support-seeking behaviour is an important skill which can help them throughout life. Where appropriate, encouraging them to self-refer for counselling within the university. Counselling on site offers an opportunity that could lead to significant lifelong benefits. Your relationship can help the advisee develop positive habits and strategies to overcome academic, social and family challenges that may impede their academic performance. Chickering and Reisser (1993) propose that development in managing emotions, developing mature interpersonal relationships, establishing identity and developing purpose and integrity are likely to ensure a student is an effective post graduate within the field of their choosing. Extenuating circumstances can take up a young adult’s time, a difficult personal relationship, for example. They can spend a lot of time thinking, discussing or agonising over aspects in their life which are new to them. This often detracts from study time and if it lasts for a lengthy period, or occurs at a crucial time in the curriculum, their academic progress can suffer significantly.

Day to day decision-making can be easy, but larger decisions that have layers of complexity may be new to the advisee. Assisting the student to identify the issues involved, generate and explore possible alternatives and weigh these up can go a long way in supporting the advisee to make a decision that is right for them as well as provide a positive foundation for future occasions.

## Tip 11 - Keep your own record of the meetings with your advisee

The adviser should make contemporary notes about the session, documenting discussion, decisions and actions, along with the student’s reactions to key points made. Whilst making these records, the adviser can note when to follow up on any points, such as any recommendations for the student to carry out. The record also offers preparation for the next session. Whilst recording the meeting, it is worth mentioning that advisers’ notes are considered an educational record and therefore subject to the university laws on data protection, confidentiality and freedom of information acts.

## Tip 12 - Consider undertaking professional development of your role as an adviser

Meetings with advisees can be very rewarding and advisers should consider the benefits to their own personal development, such as the opportunity to develop the interpersonal skills of effective listening and questioning. Reflect on whether you are doing most of the talking. Did you really listen to the advisee, or could you have asked some more probing questions? What additional information or approach might better encourage a student to take action regarding your suggestions? In addition, advisers can explore with colleagues or senior adviser management more complex situations in order to develop appropriate strategies for resolving those issues or deciding on appropriate referral pathways. For example in relation to the student who has declared a disability or a student who may be experiencing an acute mental crisis.

## Conclusions

The academic adviser should create a supportive atmosphere by demonstrating a genuine interest in the advisee. They should provide wisdom and information that is relevant and individualised. They should promote informed shared decision-making and independent thinking by assisting students in the exploration of their goals. The discussions over the duration of the course should encompass personal, academic, professional and career topics and explore the context within which the student is learning, their ability, resources and aspirations. Each adviser session will have an adviser’s agenda as well as a student’s agenda with the overall goal of supporting each and every student to reach their full potential and be proactive in preparing them for graduation. By fully engaging in this role, it can become one of the most rewarding roles for the academic.

## Take Home Messages

• The personal adviser’s role offers a golden opportunity to influence a student’s academic progression and wellbeing during their undergraduate years.
• Take time to consider developing this teaching role beyond solely academic support.
• Consider proactive advising.
• Developing an understanding of the advisee’s personal situation, their approach to studying and learning, their ability and the resources available to them will lead to adviser/advisee meetings becoming more effectively student centered.

## Notes On Contributors

Dr Margaret Bunting is a Senior Lecturer at the Norwich Medical School, University of East Anglia. She completed her Doctorate in Education in 2016, where she explored how medical students go about learning medicine. Her current role as the Director of Student Support within the medical school includes overseeing the personal advising system within the MB BS programme. She is currently on the GMC’s Health and Disability Steering Group, which is reviewing and developing the GMC guidance Welcomed and Valued: Supporting disabled learners in medical education and training.
