# Supplementary material for: Twelve tips for personal advisers to undergraduate medical students
Source: MedEdPublish (2016). 2018 Sep 5;7:198. [Version 1] doi: 10.15694/mep.2018.0000198.1 (PMC10701845; doi:10.15694/mep.2018.0000198.1)
Supplement: Peer review history

# Reviewer report: Reviewer response for version 1

**DOI:** 10.21956/mep.19651.r29305

**Published:** 2018-09-24

**Authors:** Joy Cox

**Affiliations:** 1 American University of Antigua

This review has been migrated. The reviewer awarded 4 stars out of 5 This paper provides its reader with twelve useful tips that align with personal advising best practices. The author is experienced and well-trained in the profession and this contributes to her detailed knowledge and expertise. The author outlines the responsibilities and effective advising practices of not only the adviser but also the advisee. The abstract alludes to what readers can expect from the article and encourages the reader to view advisees from a holistic perspective. The abstract mentions training for faculty advisors which was not described in the paper. I agree that preparation for each advising session is important to a successful encounter but in addition to gain students’ confidence, students need to feel welcomed in a safe and comfortable advising environment. The author outlines the importance of advisers understanding the students’ perspective by being attentive and practicing active listening. This is essential because purposeful listening helps advisers connect to the advisees and creates meaningful discussions. I concur that developing learning and study strategies and becoming a self-regulated learner is imperative in medical students’ success in medical school. Identifying students’ strengths and weaknesses is another very significant adviser behavior discussed in the paper. Since the rate, volume and the adaptation of material in medical school differs from what students might have experienced previously, the paper may be strengthened by the application of each tip specifically to medical students.

Reviewer Expertise:

NA

No decision status is available

# Reviewer report: Reviewer response for version 1

**DOI:** 10.21956/mep.19651.r29304

**Published:** 2018-09-12

**Authors:** Hui Meng Er

**Affiliations:** 1 International Medical University

This review has been migrated. The reviewer awarded 3 stars out of 5 The author has given twelve useful tips for personal advisors to undergraduate medical students based on personal experience, research on student perception and discussion with colleagues. These are relevant to universities which practise a personal advisor/advisee (or equivalent) system. To help the readers from universities that do not have such system to understand the context, some description of its objectives and how it works would be useful. For example, the Harden’s paper on the “twelve roles of a teacher” which identifies mentoring as one of the roles of a teacher could be cited. The introduction is quite brief, the background to the paper is not clear. It would be useful to include some information on the common challenges faced by personal advisors, so that the readers can relate the tips to the problems.The paper gives a comprehensive list of strategies for the personal advisors, but they should go hand-in-hand with appropriate institutional support. For example, time and effort are required from the personal advisor especially when he/she may be having a few advisees. Realistic workload and recognition should be considered. Although institutional support is not the main focus of the paper, it should be highlighted as it plays an important role in successful implementation of personal advisor/advisee system in universities.

Reviewer Expertise:

NA

No decision status is available

# Reviewer report: Reviewer response for version 1

**DOI:** 10.21956/mep.19651.r29306

**Published:** 2018-09-06

**Authors:** P Ravi Shankar

**Affiliations:** 1 American International Medical University

This review has been migrated. The reviewer awarded 4 stars out of 5 Personal advising is an important role for faculty members in most medical schools. The relationship between the adviser and the advisee is an important one and can contribute to the growth and development of both individuals. As the author rightly points out often neither the adviser nor the advisee are instructed about and are clear regarding this role. Schools may provide some training programs to faculty advisers regarding their role as advisers but many may struggle with doing justice to this role. The article by an experienced medical educator puts forward twelve practical tips for student advising and will be of interest to all medical educators and faculty members. These articles in MedEdPublish follow the structure followed for similar papers in the Medical Teacher. I found this article to be well-written and logically sequenced.

Reviewer Expertise:

NA

No decision status is available
